# Supplementary material for: The ‘Surprise’ Question in Haemato-Oncology: The Estimating Physician and Time to Death Reduce the Prognostic Uncertainty—An Observational Study
Source: Cancers (Basel). 2025 Apr 15;17(8):1326. doi: 10.3390/cancers17081326 (PMC12026296; doi:10.3390/cancers17081326)

Figure S1. Kaplan Meier survival function of patients stratified by cancer diagnosis (haematologic malignancies and solid tumours). Log rank test ($p < 0.001$).

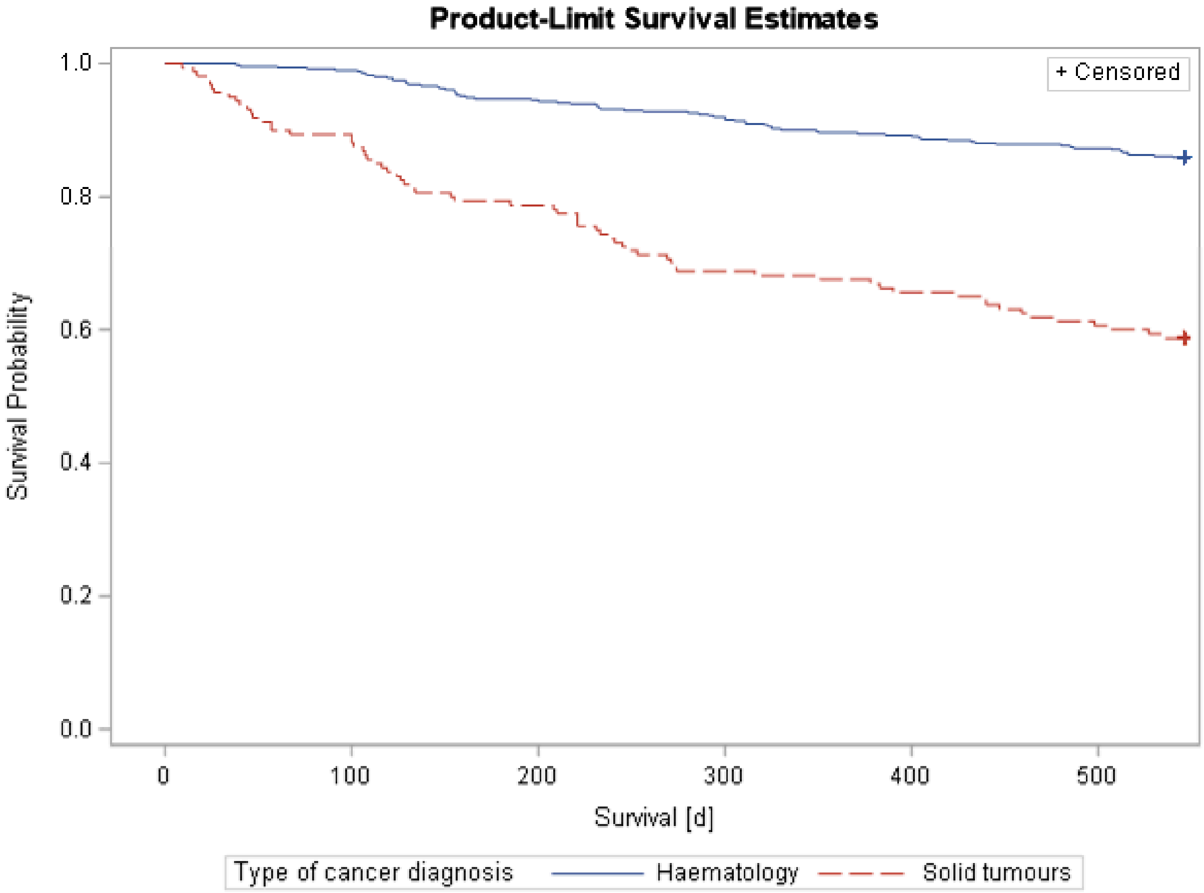

Supplement: Supplementary file 1 [file cancers-17-01326-s001.zip › cancers-3536738-supplementary.pdf]
